# The influence of norepinephrine and phenylephrine on cerebral perfusion and oxygenation during propofol–remifentanil and propofol–remifentanil–dexmedetomidine anaesthesia in piglets

**DOI:** 10.1186/s13028-018-0362-z

**Published:** 2018-02-08

**Authors:** Mai Louise Grandsgaard Mikkelsen, Rikard Ambrus, Rune Rasmussen, James Edward Miles, Helle Harding Poulsen, Finn Borgbjerg Moltke, Thomas Eriksen

**Affiliations:** 1 Department of Veterinary Clinical Sciences, University of Copenhagen, 16 Dyrlægevej, 1870 Frederiksberg C, Denmark; 2 Department of Surgical Gastroenterology C, Rigshospitalet, University of Copenhagen, 9 Blegdamsvej, 2100 Copenhagen Ø, Denmark; 3 Department of Neurosurgery, The Neuroscience Centre, Rigshospitalet, University of Copenhagen, 9 Blegdamsvej, 2100 Copenhagen Ø, Denmark; 4 Department of Neuroanaesthesia, Rigshospitalet, University of Copenhagen, 9 Blegdamsvej, 2100 Copenhagen Ø, Denmark; 5 Department of Anaesthesia, Bispebjerg and Frederiksberg Hospitals, University of Copenhagen, 23 Bispebjerg Bakke, 2400 Copenhagen NV, Denmark

**Keywords:** Cerebral oxygenation, Cerebral perfusion, Dexmedetomidine, Laser speckle contrast imaging, Licox, NIRS, Norepinephrine, Phenylephrine, Propofol, Remifentanil, Vasopressor

## Abstract

**Background:**

Vasopressors are frequently used to increase blood pressure in order to ensure sufficient cerebral perfusion and oxygenation (CPO) during hypotensive periods in anaesthetized patients. Efficacy depends both on the vasopressor and anaesthetic protocol used. Propofol–remifentanil total intravenous anaesthesia (TIVA) is common in human anaesthesia, and dexmedetomidine is increasingly used as adjuvant to facilitate better haemodynamic stability and analgesia. Little is known of its interaction with vasopressors and subsequent effects on CPO. This study investigates the CPO response to infusions of norepinephrine and phenylephrine in piglets during propofol–remifentanil and propofol–remifentanil–dexmedetomidine anaesthesia. Sixteen healthy female piglets (25–34 kg) were randomly allocated into a two-arm parallel group design with either normal blood pressure (NBP) or induced low blood pressure (LBP). Anaesthesia was induced with propofol without premedication and maintained with propofol–remifentanil TIVA, and finally supplemented with continuous infusion of dexmedetomidine. Norepinephrine and phenylephrine were infused in consecutive intervention periods before and after addition of dexmedetomidine. Cerebral perfusion measured by laser speckle contrast imaging was related to cerebral oxygenation as measured by an intracerebral Licox probe (partial pressure of oxygen) and transcranial near infrared spectroscopy technology (NIRS) (cerebral oxygen saturation).

**Results:**

During propofol–remifentanil anaesthesia, increases in blood pressure by norepinephrine and phenylephrine did not change cerebral perfusion significantly, but cerebral partial pressure of oxygen (Licox) increased following vasopressors in both groups and increases following norepinephrine were significant (NBP: P = 0.04, LBP: P = 0.02). In contrast, cerebral oxygen saturation (NIRS) fell significantly in NBP following phenylephrine (P = 0.003), and following both norepinephrine (P = 0.02) and phenylephrine (P = 0.002) in LBP. Blood pressure increase by both norepinephrine and phenylephrine during propofol–remifentanil–dexmedetomidine anaesthesia was not followed by significant changes in cerebral perfusion. Licox measures increased significantly following both vasopressors in both groups, whereas the decreases in NIRS measures were only significant in the NBP group.

**Conclusions:**

Cerebral partial pressure of oxygen measured by Licox increased significantly in concert with the vasopressor induced increases in blood pressure in healthy piglets with both normal and low blood pressure. Cerebral oxygenation assessed by intracerebral Licox and transcranial NIRS showed opposing results to vasopressor infusions.

**Electronic supplementary material:**

The online version of this article (10.1186/s13028-018-0362-z) contains supplementary material, which is available to authorized users.

## Main text

### Background

Vasopressors are frequently used to increase blood pressure in order to ensure sufficient cerebral perfusion and oxygenation (CPO) during hypotensive periods induced by e.g. anaesthesia or septic shock [1–4]. Two of the most commonly used vasopressors are norepinephrine and phenylephrine [5]. The quality of the response in cerebral blood flow (CBF) to vasopressor treatment has been shown to rely on the type of vasopressor used [3, 6, 7] and on the concurrent anaesthetic protocol [3].

Vasopressors have a well-documented systemic cardiovascular effect [8]. The primary benefit of vasopressors on CPO is believed to be due to the concomitant elevation of cerebral perfusion pressure following elevation of the systemic blood pressure [9]. However, if systemic pressure is kept within the limits of cerebral autoregulation, vasopressor treatment should have little effect on cerebral perfusion, despite an increase in blood pressure [9, 10]. Porcine cerebral arteries and veins have been reported to have dense sympathetic innervation and to be susceptible to vasopressor induced vasoconstriction in vitro [11]. Vasopressor-induced vasoconstriction has also been reported in vivo in healthy humans [12]. The sympathomimetic effect of norepinephrine is mediated by binding to both α-(α_1_) and β-(β_1_ and β_2_) adrenergic-receptors, whereas phenylephrine is a highly selective α_1_-agonist. The cerebral veins are more sensitive to sympathetic activation than cerebral arteries, and vasoconstriction are more specifically mediated by α_2_ rather than α_1_ adrenoceptors [11]. These differences may have contributed to the varying vasopressor effect on cerebral arteries versus veins observed in humans [12]. Despite the presence of α-adrenoceptors in the cerebral arteries, the vasoconstrictive effect of vasopressors on the cerebral arteries has been reported as clinically insignificant, since maximal stimulation has been shown to only reduce CBF by 5–10%, in healthy humans [10, 13].

General anaesthesia with propofol in combination with a potent opioid, such as remifentanil, has become increasingly popular [14] and the preferred total intravenous anaesthesia (TIVA) protocol in neuroanaesthesia and paediatric intensive care units [4, 15]. Propofol alone or in combination with remifentanil has been shown to preserve cerebral autoregulation in anaesthetic doses in both human [16–18] and animal studies [19, 20], and may thus be favourable in experimental studies requiring intact cerebral autoregulation. The α_2_-agonist, dexmedetomidine, has been receiving increasing attention as anaesthetic adjuvant in human anaesthesia and intensive care because of its abilities to preserve cerebral autoregulation and because of its near-ideal sedation. It has furthermore been recognized for facilitating better haemodynamic stability, analgesia and neuroprotection [15, 21–24]. In veterinary anaesthesia, α_2_-agonists have been widely used for many years as premedication, sedation and analgesia [25–27]. The hemodynamic effect of dexmedetomidine has shown to be both dose- and species dependent [28]. Dexmedetomidine has been shown to decrease systemic blood pressure in both humans and animals and to cause a generalized decrease in CBF [29–34]. This has not consistently been associated with decreased cerebral metabolic rate of oxygen [30, 31] and concern has been raised that dexmedetomidine may have the potential to cause cerebral vasoconstriction [23, 35].

The potential interactive vasoconstrictive effect of vasopressors and anaesthetics on the cerebral vasculature may be of concern in regard to CBF in hemodynamically compromised or neurocritical patients [36, 37]. The effect of vasopressors on CBF may be influenced by anaesthesia if cerebral autoregulation is preserved (intravenous anaesthesia) or impaired (inhalation anaesthesia) [3, 16–18], the latter making CBF more blood pressure-dependent with high gas concentrations [38], or when used with anaesthetics that might precondition cerebral vasoconstriction (α_2_-agonists) [39]. The systemic adrenoceptor-mediated properties of norepinephrine and phenylephrine produce different circulatory effects [40, 41]. Norepinephrine has been shown to improve myocardial function whereas phenylephrine decreases ventricular performance. In addition, norepinephrine appears to decrease microcirculatory blood flow to the abdominal organs, whereas phenylephrine does not [5]. Therefore, the choice of anaesthetic protocol in experimental animal studies should include consideration of such interactions to avoid adverse effects on CPO.

The objective of this study was to investigate the CPO response to vasopressor infusion with norepinephrine and phenylephrine during propofol–remifentanil and propofol–remifentanil–dexmedetomidine TIVA, in healthy piglets with normal and lowered blood pressure.

### Methods

#### Study design and animals

Full study details and data regarding the entire study, and results regarding the effect of dexmedetomidine on CPO have been reported elsewhere [42] and are presented in Additional file 1. The same animals were used for the study of vasopressor effect on CPO in this study.

**Fig. 1:**
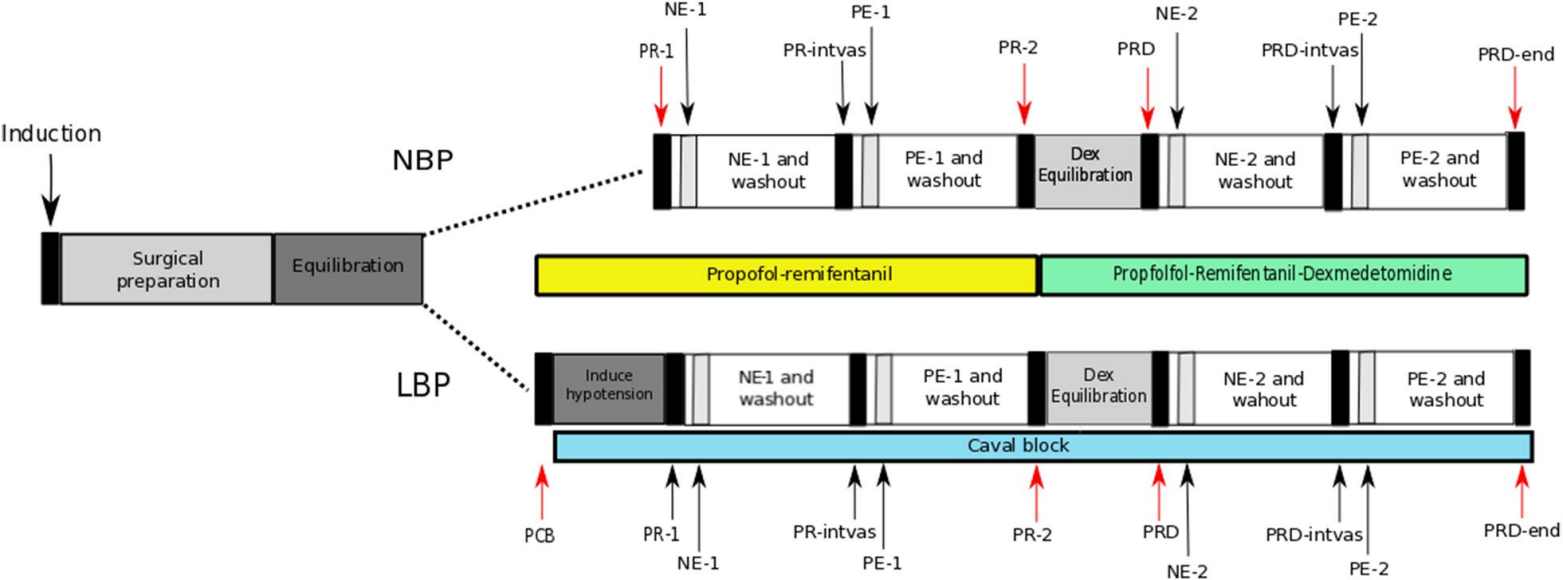
Experimental design. The experimental design and flow for the two groups (*NBP* normal blood pressure group, *LBP* low blood pressure group). Key time points are marked with arrows and vertical bars. Solid vertical bars show pre- or post-intervention baselines, where the red arrows indicate supplemental blood gas readings. Open vertical bars show vasopressor challenges. Yellow horizontal bar indicates period with propofol–remifentanil TIVA, green horizontal bar indicates period with propofol–remifentanil–dexmedetomidine, and blue horizontal bar indicates period with induced hypotension. *PCB* pre-caval block, *PR-1* baseline during propofol–remifentanil, *NE-1* norepinephrine during propofol–remifentanil, *PR-intvas* after norepinephrine and wash-out period during propofol–remifentanil, *PE-1* phenylephrine during propofol–remifentanil, *PR-2* after phenylephrine wash-out period/pre-dexmedetomidine during propofol–remifentanil, *PRD* propofol–remifentanil–dexmedetomidine, *NE-2* norepinephrine during propofol–remifentanil, *PRD-intvas* after norepinephrine and wash-out period during propofol–remifentanil–dexmedetomidine, *PE-2* phenylephrine during propofol–remifentanil–dexmedetomidine, *PRD-end* after phenylephrine and wash-out period during propofol–remifentanil–dexmedetomidine (end of experiment)

In summary: This study was designed as a non-blinded, randomized, two-arm parallel group, experimental animal trial (Fig. 1). Sixteen female slaughter piglets (Danish Landrace/Yorkshire/Duroc) with a body weight of 25–34 kg were used. In one group, low blood pressure (LBP) was induced using caval block [43, 44], whereas in the other group normal blood pressure (NBP) was maintained. All animals were subjected to the same anaesthetic protocol and did not receive premedication prior to induction on the day of the experiment.

#### Anaesthesia

General anaesthesia was induced while the animals were still in their pen to minimise stress. A dosage of 4–8 mg/kg propofol was given through a catheter placed in an auricular vein the day before the experiment. After endotracheal intubation, the animals were connected to a mechanical ventilator. End tidal CO_2_ (EtCO_2_) was maintained between 35 and 50 mmHg. Fraction of inspired oxygen (FiO_2_) was maintained around 0.8, and was centrally supplied by an in-house generator.

General anaesthesia was maintained using a TIVA protocol with separate syringe pumps (Terumo, Terufusion Syringe Pump TE-331, Belgium) for propofol (12–20 mg/kg/h) and remifentanil (20–45 µg/kg/h) TIVA. Doses were individually regulated to accomplish unresponsiveness to noxious stimuli (dewclaw pinching), with propofol doses adjusted to control anaesthetic depth (assessed as lack of movement) and remifentanil doses adjusted to eliminate responses to noxious stimuli. Once animal preparation was completed, anaesthetic doses were kept unchanged. Dexmedetomidine was supplemented after the first half of the experiment (Fig. 1) with a bolus of 1 µg/kg given over 10 min followed by a fixed dose of 0.7 µg/kg/h iv.

#### Surgical preparation and instrumentation

All invasive procedures were conducted after infiltration with a mixture of lidocaine and bupivacaine. After surgical cut down, the femoral artery was cannulated for invasive blood pressure monitoring and intermittent blood collection for blood gas analysis. An eight French balloon-tipped catheter was placed in the femoral vein in all animals, with the balloon positioned in the caudal vena cava just below the heart. A urinary catheter with a closed collecting bag was placed to prevent bladder distension. Isotonic glucose solution was administered throughout the experiment at 2.5 mL/kg/h. A multiparametric bedside monitor recorded haemodynamic and respiratory variables every 30 s, and data were transferred to a personal computer using Datex-Ohmeda S/5™ Collect software (GE Healthcare, Helsinki, Finland). The collected variables were pulse rate, and mean arterial blood pressure (MAP), body temperature (oesophageal probe), fractionated inspired oxygen (FiO_2_), and EtCO_2_ (Additional file 1). Electrocardiogram and peripheral oxygen saturation by pulse oximetry (S_p_O_2_) measured on the tail or the lower lip were monitored for continuous assessment.

#### Cerebral perfusion and oxygenation (CPO) measures

A circular craniotomy (20–30 mm) was performed over the right parietal lobe with a 5 mm craniotome, and dura was removed. A laser speckle contrast imaging (LSCI) camera (MoorFLPI-2, Moor Instruments, Devon, UK) was used to measure cerebral perfusion semi quantitatively in laser speckle perfusion units (LSPU). The position of the head of the animal remained unchanged throughout the experiment and the focus distance was 25 cm. Cerebral partial pressure of oxygen (P_br_O_2_) was measured by an intracerebral Clark-type probe (Licox, Integra LifeSciences, New Jersey, USA) which was placed 25 mm subdurally into the white matter and secured to the craniotomy edge with bone wax. Non-invasive measurement of cerebral oxygen saturation (S_br_O_2_) was obtained by near infrared spectroscopy (NIRS) (Invos 5100, Covidien/Medtronic, Minneapolis, USA). A sensor was attached to the skin of the forehead on the left side of the animal, on the contralateral side to the Licox probe and the LSCI camera, and isolated from external light.

#### Experimental protocol

After instrumentation the Licox probe was equilibrated for a period of 2 h or until P_br_O_2_ > 25 mmHg, and followed by baseline data collection (PR-1–NBP and PCB–LBP) for all animals (Fig. 1). The blood pressure was lowered by caval block in the LBP group (by inflation of the balloon catheter in the vena cava) until a stable MAP of 50–60 mmHg was achieved, and an additional baseline was recorded in this group (PR-1–LBP). The caval block was maintained throughout the experiment for the LBP group, and was not subjected to further adjustments.

Vasopressor intervention followed the same sequence (norepinephrine followed by phenylephrine) in both groups and was repeated after initiation of dexmedetomidine infusion. Baseline recordings were obtained before and after each intervention or washout period (Fig. 1). The standard 30 min washout periods were conservatively chosen to allow the blood pressure and the CPO measures to return to baseline values between vasopressor interventions, and were chosen based on the longest reported clinical effect time of 15 min for phenylephrine [45]. Norepinephrine (1 mg/mL Noradrenalin “SAD”, Amgros I/S, Copenhagen, Denmark) was administered by bolus (100 µg) and followed by infusion of (0.6–2.0 µg/kg/min) to a target effect of either MAP 130–140 mmHg or 100% increase in MAP (primarily for the LBP group) from the baseline. Similarly, phenylephrine (1 mg/mL Metaoxedrin “SAD”, Amgros I/S, Copenhagen, Denmark) was administered by bolus (200 µg) and followed by infusion of 5.0–13.5 µg/kg/min to a target effect of either MAP 130–140 mmHg or 100% increase (primarily for the LBP group) in MAP from the baseline. During the experiment, arterial blood samples were collected at PCB, PR-1, PR-2, PRD and PRD-end for blood gas and acid–base evaluation (Fig. 1 and Additional file 1). All animals were euthanized with pentobarbital IV at the end of the experiment.

#### Statistics

Statistical analysis was performed using SPSS 24.0 software (IBM^®^ SPSS^®^ Statistics for Mac, IBM Corp. ©, Armonk, NY, USA), and Microsoft^®^ Excel^®^ for Mac 2011 version 14.3.9 (2010 Microsoft Corporation). Non-parametric statistical tests were used, since normal distribution of data could not be assumed due to the small sample size. Data were reported as medians and range (min–max), and differences between groups were analysed by the independent-samples median test. Median changes for all outcome variables were reported with 95% confidence intervals (95% CI) using Hodges-Lehmann estimates where appropriate. Primary (Licox, NIRS and LSCI) and secondary (MAP, pulse rate and EtCO_2_) outcome variables at time points PR-1, NE-1, PR-intvas, PE-1, PRD, NE-2, PRD-intvas and PE-2 were compared using Friedman’s ANOVA with post hoc pairwise comparisons. Significance levels for the four comparisons of interest (before vasopressor and during vasopressor administration) were controlled using Holm–Bonferroni’s correction before reporting. Significance was set at the 5% level.

A sample size of 16 animals, divided into two groups of 8, was calculated using conservative estimates based on earlier studies [46] with expected power of 80% in detecting a minimum of 30% difference in MAP with a two-tailed significance level of 5% after supplementation of dexmedetomidine.

### Results

All 16 animals completed the experimental protocol. Data from three (NBP group n = 2, LBP group n = 1) piglets were excluded from analysis. One piglet developed signs of brain oedema with a severe reduction in CPO following craniotomy (LBP group). In the NBP group, one piglet had persistently and unexplainably high pulse rate, EtCO_2_, P_a_CO_2_ and a low pH, which were expected to produce an atypical CPO response. The other piglet was excluded due to technical difficulties.

The remaining 13 piglets reached the target MAP with vasopressor administration of > 130 mmHg or a 100% increase over pre-treatment MAP. The animals of the two groups revealed no significant demographic differences, nor were any significant differences revealed in anaesthesia time, preparation time, anaesthetic doses, baseline P_br_O_2_ or LSPU measurements after equilibration [42]. Additionally, there was found no significant difference between the two groups in S_br_O_2_ measured by NIRS (P = 0.59), which were 65% (range 59–72) in the LBP group and 62% (range 51–70) in the NBP group at PCB and PR-1 respectively. Both groups reached normal P_br_O_2_ and S_br_O_2_ levels [47, 48] after equilibration.

#### Vasopressor effects under propofol–remifentanil anaesthesia

Norepinephrine administration significantly increased MAP in both groups [LBP: P = 0.002, median increase 61 mmHg (95% CI 47; 71), NBP: P = 0.009, median increase 51 mmHg (95% CI 39; 66)]. Following washout MAP was not significantly different to pre-treatment levels in either group (P = 1) with median differences of 1 mmHg (95% CI − 8; 11) for LBP and 7 mmHg (95% CI − 6; 17) for NBP.

**Fig. 2:**
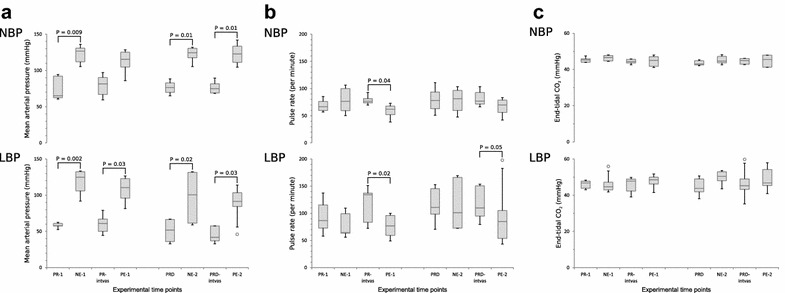
Boxplots of haemodynamic data at baselines and vasopressor interventions. Absolute data presented as boxplots with median and interquartile range. Open circles indicate outliers. All comparisons with significant changes between interventions and the immediate pre-intervention baselines are marked with horizontal solid lines and exact P values are noted. **a** mean arterial pressure (MAP), **b** pulse rate, **c** end-tidal carbon dioxide (EtCO_2_). For all variables, the results of the normal blood pressure (NBP) are presented on the top chart and results from low blood pressure (LBP) group are presented at the bottom chart. The x-axis represents the experimental time-points, and the y-axis shows the names and units of the individual variables. *NE-1/PE-1* norepinephrine/phenylephrine infusion during propofol–remifentanil, *NE-2/PE-2* norepinephrine/phenylephrine infusion during propofol–remifentanil–dexmedetomidine, *PCB* pre-caval block (only LBP), *PR-1* baseline before interventions (after caval block in LBP), *PR-intvas* after NE-1 and 30-min washout, *PR-2* after PE-1 and 30-min washout, *PRD* baseline after infusion start of dexmedetomidine, *PRD-intvas* after NE-2 and 30-min washout, *PRD-end* after PE-2 and 30-min washout

Phenylephrine administration significantly (P = 0.03) increased MAP in the LBP group [median increase 47 mmHg (95% CI 32; 66)] but not in the NBP group [P = 0.13, median increase 33 mmHg (95% CI 14; 55)] (Fig. 2a).

Pulse rate decreased significantly in both groups following phenylephrine (LBP: P = 0.02, NBP: P = 0.04) but not norepinephrine (LBP: P = 0.4, NBP: P = 0.6) (Fig. 2b).

**Fig. 3:**
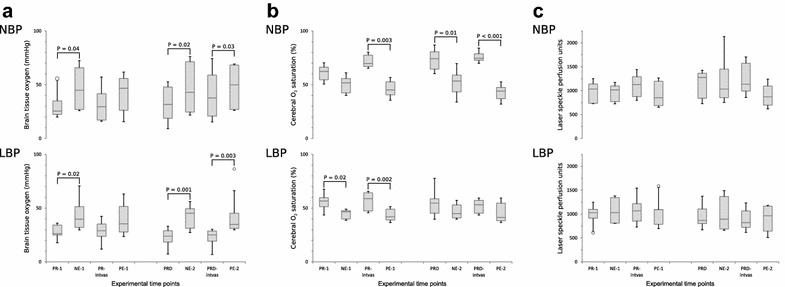
Boxplots of CPO data at baselines and vasopressor interventions. Absolute data presented as boxplots with median and interquartile range. Open circles indicate outliers. All comparisons with significant changes between interventions and the immediate pre-intervention baselines are marked with horizontal solid lines and exact p-values are noted. **a** Licox, **b** near infrared spectroscopy (NIRS), **c** laser speckle contrast imaging (LSCI). For all variables, the results of the normal blood pressure (NBP) are presented on the top chart and results from low blood pressure (LBP) group are presented at the bottom chart. The x-axis represents the experimental time-points, and the y-axis shows the names and units of the individual variables. *NE-1/PE-1* Norepinephrine/phenylephrine infusion during propofol–remifentanil, *NE-2/PE-2* Norepinephrine/phenylephrine infusion during propofol–remifentanil–dexmedetomidine, *PCB* pre-caval block (only LBP), *PR-1* baseline before interventions (after caval block in LBP), *PR-intvas* after NE-1 and 30-min washout, *PR-2* after PE-1 and 30-min washout, *PRD* baseline after infusion start of dexmedetomidine, *PRD-intvas* after NE-2 and 30-min washout, *PRD-end* after PE-2 and 30-min washout

Cerebral partial pressure of oxygen (P_br_O_2_) increased significantly following norepinephrine (P = 0.02) but not phenylephrine (P = 0.06) in the LBP group (median increases 14 mmHg (95% CI 6; 26) and 11 mmHg (95% CI 2; 23), respectively). A similar response was observed in the NBP group with a significant increase following norepinephrine [P = 0.04, median increase 17 mmHg (95% CI 5; 33)] but not phenylephrine [P = 0.2, median increase 8 mmHg (95% CI 2; 26)] (Fig. 3a).

In contrast, cerebral oxygen saturation (S_br_O_2_) fell significantly in the LBP group following both norepinephrine and phenylephrine [P = 0.02, median decrease − 11% (95% CI − 20; − 3) and P = 0.002, median decrease − 15% (95% CI − 23; − 7), respectively] and in the NBP group S_br_O_2_ fell significantly following phenylephrine [P = 0.003, median decrease − 24% (95% CI − 31; − 22)] but not norepinephrine [P = 0.2, median decrease − 10% (95% CI − 16; − 6)] (Fig. 3b). LSCI measurements (LSPU) did not exhibit a clear trend to increase or decrease, and no significant changes were observed following vasopressor administration (Fig. 3c).

While EtCO_2_ readings showed increased variability in the LBP group compared to the NBP group, neither group’s EtCO_2_ readings responded significantly to vasopressor treatment (Fig. 2c).

#### Vasopressor effects under propofol–remifentanil–dexmedetomidine anaesthesia

Norepinephrine administration significantly increased MAP in both groups [LBP: P = 0.02, median increase 46 mmHg (95% CI 17; 81), NBP: P = 0.01, median increase 48 mmHg (95% CI 33; 57)]. Following washout MAP was not significantly different to pre-treatment levels in either group (P = 1.0) with median differences of − 6 mmHg (95% CI − 12; 3) for LBP and − 1 mmHg (95% CI − 5; 4) for NBP.

Phenylephrine administration significantly increased MAP in both groups [LBP: P = 0.03, median increase 44 mmHg (95% CI 20; 66), NBP: P = 0.01, median increase 48 mmHg (95% CI 34; 62)] (Fig. 2a).

Pulse rate did not alter significantly in either group with either vasopressor in the NBP group, but fell significantly following phenylephrine in the LBP group [P = 0.05, median decrease − 25 (95% CI − 66; 34)] (Fig. 2b).

Cerebral partial pressure of oxygen (P_br_O_2_) increased significantly in the LBP group following both norepinephrine and phenylephrine [P = 0.001, median increase 18 mmHg (95% CI 11; 28) and P = 0.003, median increase 16 mmHg (95% CI 7; 42)]. In the NBP group, a similar response was seen with significant increases seen following norepinephrine [P = 0.02, median increase 13 mmHg (95% CI 6; 23)] and phenylephrine [P = 0.03, median increase 10 mmHg (95% CI − 2; 16)] (Fig. 3a).

Cerebral oxygen saturation (S_br_O_2_) did not fall significantly in the LBP group following either norepinephrine [P = 0.08, median decrease − 7% (95% CI − 20; 1)] or phenylephrine [P = 0.14, median decrease − 7% (95% CI − 17; − 1)]. In contrast, the NBP group experienced significant decreases following both norepinephrine [P = 0.01, median decrease − 21% (95% CI − 26; − 18)] and phenylephrine [P < 0.001, median decrease − 33% (95% CI − 37; − 30)] (Fig. 3b). As for the NBP group, LSCI did not measure any significant changes in cerebral perfusion (LSPU) following vasopressor administration (Fig. 3c).

The LBP group continued to exhibit increased variability in EtCO_2_ readings compared to the NBP group, but no significant changes were observed due to vasopressor administration (Fig. 2c).

### Discussion

Cerebral partial pressure of oxygen (P_br_O_2_) was found to increase during vasopressor challenges when assessed by Licox, while cerebral oxygen saturation (S_br_O_2_) decreased when assessed by NIRS. Cerebral perfusion (LSPU) was not found to change significantly in concert with the vasopressor induced increases in MAP. This pattern of findings was similar in both groups and during both TIVA protocols (Fig. 3c), suggesting that the cerebral autoregulation did remain intact throughout the experiment. This is consistent with the findings of Bruins et al. [3] indicating preserved cerebral autoregulation during TIVA (midazolam and fentanyl), in contrast to inhalation anaesthesia (isoflurane-based) where autoregulation was impaired.

The increase in P_br_O_2_ during norepinephrine or phenylephrine has been previously reported in pigs with both uninjured brains [49] and traumatic brain injury [7]. In the present study, the significant increase in P_br_O_2_ during vasopressor infusion was not accompanied by a concurrent increase in perfusion, suggesting that changes in P_br_O_2_ do not simply reflect changes in CBF [50]. Decreasing S_br_O_2_ with increasing P_br_O_2_ was observed during vasopressor infusions, in both the propofol–remifentanil and propofol–remifentanil–dexmedetomidine TIVA groups (Fig. 3a, b). These results are consistent with findings in human NIRS studies, where a decrease in S_br_O_2_ in response to blood pressure elevation by norepinephrine [51] or phenylephrine [6, 12, 52, 53] has been reported. Human studies of NIRS have also reported increase in S_br_O_2_ after nitroprusside induced blood pressure decrease and decrease in S_br_O_2_ in response to vasopressor induced increase in blood pressure [54, 55]. Some authors speculated that this response in cerebral oxygen saturation was part of a normal cerebral autoregulatory response [54], while others have questioned the validity of NIRS technology and suggested that it primarily reflects skin perfusion rather than cerebral oxygenation [12, 37, 56–59]. Cerebral oxygen saturation values (S_br_O_2_) and cerebral partial pressure of oxygen values (P_br_O_2_) are not directly comparable in absolute values, since NIRS reflects levels of oxygen-saturated haemoglobin in the venous, capillary and arterial blood [60] and Licox has been described as a measure of “the pool of oxygen” that accumulates in the brain tissue and thus reflects the balance between oxygen delivery, diffusion and consumption [50, 61, 62]. The distribution ratio of arteries versus veins in the cerebral cortex is approximately 30:70, and NIRS therefore predominantly reflects the cerebral venous oxygen reserve [60, 63]. Transcranial assessment of the cerebral oxygen saturation in piglets will furthermore depend on factors like skull thickness and pneumatisation of the frontal sinus, since NIRS has limited penetration. Due to the size and age of the animals used in this study, the pneumatisation of the large frontal sinus, which starts at approximately 3–4 months of age in domestic swine [64], was not expected to affect the results. NIRS purportedly reflects S_br_O_2_ in the grey matter of the cerebral cortex, whereas Licox measures P_br_O_2_ in the less metabolically active white matter of the CNS, areas with different metabolic activity and blood flow [9, 65, 66].

The differences in cerebral oxygenation assessment by Licox and NIRS could indicate that vasopressor treatment affects the cortex and the white matter differently. Whether this is a normal response attributable to a preserved cerebral autoregulation [54] or related to limitations of the methods used remains unanswered. Since cutaneous vessels predominantly have α-adrenergic innervation, treatment with vasopressors having high affinity for α-adrenergic receptors (such as norepinephrine and phenylephrine), results in vasoconstriction [67] and decreases in skin blood flow. Thus, decreases in cerebral oxygen saturation values during vasopressor infusion could therefore be a reflection of extra-cranial rather than cerebral oxygen saturation.

In summary, the CPO response to vasopressor challenge in piglets was found to be qualitatively similar for the two TIVA protocols used, and the concern regarding the potential additive vasoconstrictive effect of dexmedetomidine during vasopressor infusions could not be confirmed in this study.

#### Strengths and limitations

The current study was strengthened by omission of premedication on the day of experiment, by avoiding a possible vasoactive effect of premedication on CPO, and by using animals of the same sex and with a narrow age span since both age and gender may influences CBF in porcine models [68–70]. The results from this animal study best translates into children with neurodevelopmental maturity of approximately 10 months of age [71, 72] and translation to other age groups should be made with caution [69, 73].

We compared regional with focal CPO measures and at contralateral brain sites in animals undergoing craniotomy, which limits derived conclusions regarding global CPO status and regarding CPO in animals not undergoing craniotomy. Another potential limitation was the possibility that the initial norepinephrine infusion could have affected the physiological and cerebral response to the subsequent phenylephrine infusions. However, the vasopressors were given in the same sequence in all the animals, and any potential preconditioning was therefore expected to be similar in the entire group of animals. The supra clinical target increases in blood pressure and infusion doses of norepinephrine and phenylephrine in this study, were set to ensure an effect on CPO, if present, while retaining the animals within the expected blood pressure range for intact cerebral autoregulation. The doses though, were comparable to doses used in other animal studies [7, 74]. This intensive approach to elevate blood pressure and subsequently CPO, when compared to having used more moderate targets and vasopressor doses, may have increased the risk for type I errors. Furthermore, the design of the study was set up to illustrate haemodynamic influences in the normal as well as in hemodynamically compromised patients without initial brain pathologies. Extrapolation to situations with concurrent brain pathology should be made with caution since neurophysiology and cerebral autoregulation may be different. The animals were subjected to prolonged anaesthesia, which for some animals lasted up to 10–11 h, and a mild to moderate hypercapnia was observed in all animals throughout the experiment [42] (Additional file 1). The resulting respiratory acidosis would make the autoregulatory plateau narrower, and thereby the cerebral perfusion more sensitive to changes in blood pressure. This was however not evident from the results of the current study, since no significant variations in cerebral perfusion could be detected. The increased levels of P_a_CO_2_ (represented by EtCO_2_ in this study) might however have increased the variability of the perfusion data, which would make the non-significant findings expected. In general, P_br_O_2_ showed greater variability and a tendency to increase in the NBP group than in the LBP group, especially after addition of dexmedetomidine. Finally, the supplemented inspiratory oxygen levels were also relatively high in this study, with a FiO_2_ of 0.8 for all animals (Additional file 1). The FiO_2_ did not differ throughout the experiment, and is therefore not expected to influence the relative changes in P_br_O_2_ of this study. It cannot be excluded that the CPO response to changes in MAP observed in this study could be different at lower FiO_2_.

Non-significant P values following Holm-Bonferroni correction were noted for several parameters despite their confidence intervals for the change in medians not including zero. The small sample size and large variability for some parameters (P_br_O_2_, LSPU, pulse rate) increased the risk of type II error. Confidence intervals were therefore reported for both significant and non-significant changes, permitting a more nuanced interpretation.

### Conclusions

Cerebral partial pressure of oxygen measured by Licox increased significantly in concert with the vasopressor induced increases in blood pressure in healthy piglets with both normal and low blood pressure. Cerebral oxygenation assessed by intracerebral Licox and transcranial NIRS showed opposing results to vasopressor infusions. The CPO responses, induced by norepinephrine and phenylephrine, were shown to be qualitatively similar during both propofol–remifentanil and propofol–remifentanil–dexmedetomidine TIVA.

## Additional file

**Additional file 1.** Table of experimental data: Cerebral perfusion and oxygenation readings, physiological and haemodynamic data, blood gas data, and anaesthesia time at all time points throughout the experiment. PCB: Pre-Caval block; PR-1: baseline during propofol-remifentanil; NE-1: Norepinephrine during propofol-remifentanil; PR-intvas: after norepinephrine and wash-out period during propofol-remifentanil; PE-1: Phenylephrine during propofol-remifentanil; PR-2: after phenylephrine wash-out period/pre-dexmedetomidine during Propofol-remifentanil; PRD: Propofol-remifentanil-dexmedetomidine; NE-2: Norepinephrine during propofol-remifentanil; PRD-intvas: after norepinephrine and wash-out period during propofol-remifentanil-dexmedetomidine; PE-2: Phenylephrine during propofol-remifentanil-dexmedetomidine; PRD-end: after phenylephrine and wash-out period during propofol-remifentanil-dexmedetomidine (end of experiment); NIRS: Near infra red spectroscopy; LSCI: Laser speckle contrast imaging; MAP: mean arterial pressure; EtCO_2_: End-tidal carbon dioxide; FiO_2_: Fraction of inspired oxygen; (T): data corrected for body temperature; PaCO_2_: Partial pressure of arterial carbon dioxide; PaO_2_: Partial pressure of arterial oxygen; HCO_3_: Hydrogen bicarbonate; Hct: Haematocrit; THbc: Total haemoglobin concentration.

## Abbreviations

CBF: cerebral blood flow
CPO: cerebral perfusion and oxygenation
CPP: cerebral perfusion pressure
EtCO_2_: end-tidal carbon dioxide
FiO_2_: fractionated inspired oxygen
LBP: low blood pressure group
LSCI: laser speckle contrast imaging
LSPU: laser speckle perfusion unit
Max: maximum
Min: minimum
MAP: mean arterial pressure
NBP: normal blood pressure group
P_a_CO_2_: arterial partial pressure of carbon dioxide
P_a_O_2_: arterial partial pressure of oxygen
P_br_O_2_: cerebral oxygenation (partial pressure of oxygen in brain tissue)
PCB: pre-caval block (time point)
PR-n: propofol/remifentanil TIVA (n: time-point number)
PRD: propofol/remifentanil TIVA + dexmedetomidine CRI (time point)
S_br_O_2_: cerebral oxygen saturation
SpO_2_: peripheral oxygen saturation by pulse oximetry
TIVA: total intravenous anaesthesia
