# Supplementary material for: The influence of norepinephrine and phenylephrine on cerebral perfusion and oxygenation during propofol–remifentanil and propofol–remifentanil–dexmedetomidine anaesthesia in piglets
Source: Acta Vet Scand. 2018 Feb 8;60:8. doi: 10.1186/s13028-018-0362-z (PMC5806235; doi:10.1186/s13028-018-0362-z)
Supplement: Supplementary file 1 — Additional file 1. Table of experimental data: Cerebral perfusion and oxygenation readings, physiological and haemodynamic data, blood gas data, and anaesthesia time at all time points throughout the experiment. PCB: Pre-Caval block; PR-1: baseline during propofol-remifentanil; NE-1: Norepinephrine during propofol-remifentanil; PR-intvas: after norepinephrine and wash-out period during propofol-remifentanil; PE-1: Phenylephrine during propofol-remifentanil; PR-2: after phenylephrine wash-out period/pre-dexmedetomidine during Propofol-remifentanil; PRD: Propofol-remifentanil-dexmedetomidine; NE-2: Norepinephrine during propofol-remifentanil; PRD-intvas: after norepinephrine and wash-out period during propofol-remifentanil-dexmedetomidine; PE-2: Phenylephrine during propofol-remifentanil-dexmedetomidine; PRD-end: after phenylephrine and wash-out period during propofol-remifentanil-dexmedetomidine (end of experiment); NIRS: Near infra red spectroscopy; LSCI: Laser speckle contrast imaging; MAP: mean arterial pressure; EtCO2: End-tidal carbon dioxide; FiO2: Fraction of inspired oxygen; (T): data corrected for body temperature; PaCO2: Partial pressure of arterial carbon dioxide; PaO2: Partial pressure of arterial oxygen; HCO3: Hydrogen bicarbonate; Hct: Haematocrit; THbc: Total haemoglobin concentration. [file 13028_2018_362_MOESM1_ESM.docx]

Cerebral perfusion and oxygenation readings, physiological and haemodynamic data, blood gas data, and anaesthesia time at all time points throughout the experiment. PCB: Pre-Caval block; PR-1: baseline during propofol-remifentanil; NE-1: Norepinephrine during propofol-remifentanil; PR-intvas: after norepinephrine and wash-out period during propofol-remifentanil; PE-1: Phenylephrine during propofol-remifentanil; PR-2: after phenylephrine wash-out period/pre-dexmedetomidine during Propofol-remifentanil; PRD: Propofol-remifentanil-dexmedetomidine; NE-2: Norepinephrine during propofol-remifentanil;PRD-intvas: after norepinephrine and wash-out period during propofol-remifentanil-dexmedetomidine; PE-2: Phenylephrine during propofol-remifentanil-dexmedetomidine; PRD-end: after phenylephrine and wash-out period during propofol-remifentanil-dexmedetomidine (end of experiment); NIRS: Near infra red spectroscopy; LSCI: Laser speckle contrast imaging; MAP: mean arterial pressure; EtCO_2_: End-tidal carbon dioxide; FiO_2_: Fraction of inspired oxygen; (T): data corrected for body temperature; PaCO_2_: Partial pressure of arterial carbon dioxide; PaO_2_: Partial pressure of arterial oxygen; HCO_3_: Hydrogen bicarbonate; Hct: Haematocrit; THbc: Total haemoglobin concentration.
